# Temperature Field-Assisted Ultraviolet Nanosecond Pulse Laser Processing of Polyethylene Terephthalate (PET) Film

**DOI:** 10.3390/mi12111356

**Published:** 2021-11-02

**Authors:** Jun Xu, Youmin Rong, Weinan Liu, Tian Zhang, Guoqiang Xin, Yu Huang, Congyi Wu

**Affiliations:** 1 State Key Lab of Digital Manufacturing Equipment and Technology, Huazhong University of Science and Technology (HUST), Wuhan 430074, China; d202080271@hust.edu.cn (J.X.); rym@hust.edu.cn (Y.R.); wnliu@hust.edu.cn (W.L.); tianz@hust.edu.cn (T.Z.); gqxin@hust.edu.cn (G.X.); yuhuang_hust@hust.edu.cn (Y.H.); 2 School of Mechanical Science and Engineering, Huazhong University of Science and Technology (HUST), Wuhan 430074, China

**Keywords:** laser processing, PET film, transparent polymer, temperature field, ultraviolet nanosecond pulse laser, laser photothermal ablation

## Abstract

Understanding the mechanism of and how to improve the laser processing of polymer films have been important issues since the advent of the procedure. Due to the important role of a photothermal mechanism in the laser ablation of polymer films, especially in transparent polymer films, it is both important and effective to adjust the evolution of heat and temperature in time and space during laser processing by simply adjusting the ambient environment so as to improve and understand the mechanism of this procedure. In this work, studies on the pyrolysis of PET film and on temperature field-assisted ultraviolet nanosecond (UV-ns) pulse laser processing of polyethylene terephthalate (PET) film were performed to investigate the photothermal ablation mechanism and the effects of temperature on laser processing. The results showed that the UV-ns laser processing of PET film was dominated by the photothermal process, in which PET polymer chains decomposed, melted, recomposed and reacted with the ambient gases. The ambient temperature changed the heat transfer and temperature distribution in the laser processing. Low ambient temperature reduced the thermal effect and an increase in ambient temperature improved its efficiency (kerf width: 39.63 μm at −25 °C; 48.30 μm at 0 °C; 45.81 μm at 25 °C; 100.70 μm at 100 °C) but exacerbated the thermal effect.

## 1. Introduction

Polymer films are widely applied in electronic devices [1], flexible devices [2], energy devices [3], etc. [4,5], due to their unique physical and chemical properties. Thus, high-precision processing methods for polymer films have received increasing attention. In particular, the rapid development of large-scale integrated circuits and various functional elements has created higher requirements for the processing precision, quality and efficiency of polymer films. Laser processing, as a non-contact processing method, has received growing attention due to its adaptability, low divergence and ability to render the selective removal of polymers with high spatial resolution [6,7], and it is widely applied in the processing of a variety of materials such as metals, glasses, polymers, etc. [8,9,10,11,12] Therefore, the high-precision laser processing of polymer films has attracted much attention due to its unique advantages [12]. In past decades, many polymers have been studied for laser micromachining such as polyethylene terephthalate (PET) [13], polyimide (PI) [14], polydimethylsiloxane (PDMS) [15], polymethyl methacrylate (PMMA) [16], polytetrafluoroethylene (PTFE) [17], etc. [4,18] However, questions regarding the improvement of the precision, quality and efficiency of laser processing, as well as the laser ablation process and mechanism, still prevail. 

According to the principles of laser processing and previous research,, there are three ways to improve the procedure by changing the interactions between lasers and materials: (1) adjusting the parameters of lasers, such as power, wavelength, pulse width, etc. [19,20] (2) adjusting the physical or chemical properties of materials, such as absorption coefficient, reflectivity, thermal conductivity, etc. [21,22,23] (3) adding physical fields or changing the external chemical environment such as temperature field, electromagnetic field, supersonic wave or specific chemical solutions [24,25]. Adjusting the laser parameters is an effective method to improve processing quality, but its effectiveness is limited in certain materials. Furthermore, although changing the material properties can bring some improvement, usually this change is irreversible and difficult for the formed polymer films. Compared with the above two methods, adding physical fields or changing the external chemical environment is more simple, effective and widely applicable.

The laser ablation process of polymer films is a combination of photothermal and photochemical processes [26]. Between these two mechanisms, the photothermal mechanism plays an important role in laser processing, especially for transparent polymer films with poor absorption. Therefore, the absorption, conversion and transfer of heat, as well as the change and distribution of temperature, affect the ablation and decomposition of polymer films during laser processing in important ways. This is key to correctly managing the evolution of heat and temperature in time and space during the laser processing of polymer films via simple adjustments to the ambient environment, so as to improve the procedure. It also helps to understand the process and mechanism of the laser processing of polymer films.

In this study, an external temperature field was applied to assist the laser processing of polymer films to investigate the effects of ambient temperature on the procedure, which made the laser ablation mechanism clearer and more understandable. The selected experimental material was PET film, which is widely used in both the electronics industry and daily life [27,28]. The applied laser was an ultraviolet (355 nm) nanosecond (UV-ns) pulse laser due to it being less expensive and possessing an appropriate wavelength for polymer. Firstly, thermogravimetric analysis (TGA), thermogravimetric analysis, Fourier transform infrared spectroscopy (TGA-FTIR), pyrolysis–gas chromatography-mass spectrometry (GC-MS) and Raman spectrometry were performed to study the thermal decomposition process and laser photothermal ablation mechanism of PET films. Secondly, laser processing experiments (single-spot ablation and line ablation) of PET films at various ambient temperatures (−25 °C, 0 °C, 50 °C, 75 °C, 100 °C) were performed to investigate the effects of ambient temperature on the morphology and geometric characteristics (size, processing area, heat affected zone (HAZ)) of features (hole and kerf) so as to study the influencing mechanism of ambient temperature on the laser processing of PET film. The results showed that the laser processing of PET film was dominated by the photothermal decomposition process, in which PET polymer chains decomposed, melted, recomposed and reacted with the ambient gases. Changes in ambient temperature affected the laser processing of PET film: an increase in ambient temperature changed the heat transfer and temperature distribution during the laser processing, while a lower ambient temperature reduced the thermal effect. Increasing ambient temperature also improved the efficiency (kerf width: 39.63 μm at −25 °C; 45.81 μm at 25 °C; 100.70 μm at 100 °C) but exacerbated the thermal effect. This work provides effective methods to study the laser processing mechanism of polymer films, as well as an approach to improve this procedure.

## 2. Materials and Methods

### 2.1. Materials

The raw material that was processed was commercial transparent PET film (Thickness: 0.2 μm), supplied by Xilu Photoelectricity Technic CO., LTD (Shenzhen, China).

### 2.2. Laser Processing System

**Figure 1:**
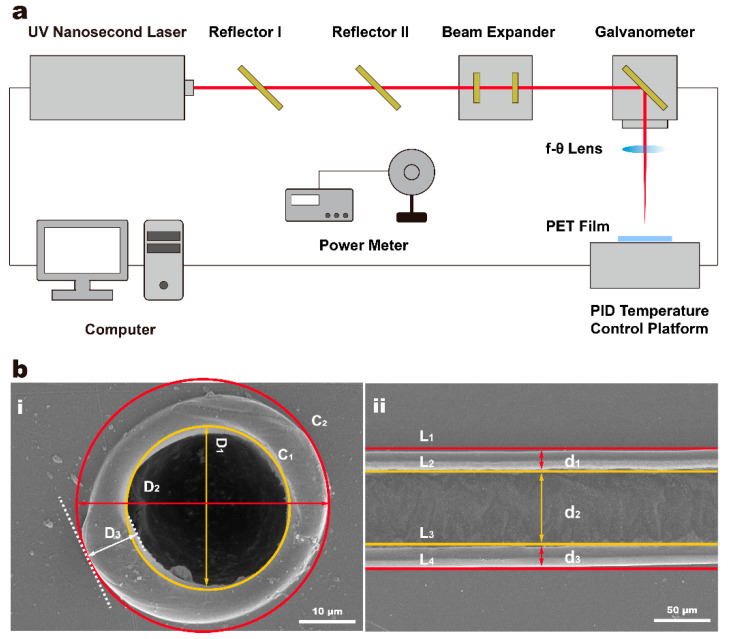
The Schematic diagram of the laser processing of PET film (**a**), and the processing quality evaluation (**b**) of different features (hole (i) and kerf(ii)).

The laser processing system used was an experimental platform built by the team. As shown in Figure 1a, the laser processing system consisted of an ultraviolet nanosecond (UV-ns) laser (Poplar-355-15A5, Wuhan Huaray Precision Laser Co. Ltd., Wuhan, China), two reflectors, a three-dimensional scanning galvanometer (intelliSCAN 14, Scanlab, Puchheim, Germany), a beam expander, a proportion–integral–differential (PID) temperature control platform and a computer. The output power of the laser was controlled by adjusting laser repetition frequency and measured with a power meter. The specific parameters of the laser processing system are listed in Table 1. PET film samples were placed on the PID temperature control platform, the surface temperature of which was regulated by either ceramic heater heating or liquid nitrogen cooling. UV-ns laser, scanning galvanometer and the PID temperature control platform were controlled by computer so that laser processing parameters (laser power, scanning speed and ambient temperature) could be adjusted.

### 2.3. Experimental Design

Photothermal ablation and thermal effects play important roles in laser processing. Heat generation and transfer can be regulated by adjusting processing parameters (laser power, scanning speed, scanning times, etc.), material properties or environmental conditions. In our experimental design, in order to investigate the influences of ambient temperature on heat generation and transfer during laser processing, single-point and line laser processing were performed. In these experiments, processing parameters (laser power, repetition frequency, duration time, scanning times, etc.) were constant and the effects of ambient temperature were investigated by temperature gradient experiments. Table 2 shows the levels of ambient temperature and processing parameters in the single-point and line laser processing experiments.

Based on the typical features (hole and kerf) of PET film samples after laser processing, processing quality evaluation methods were established. As shown in Figure 1b, in the single-point laser processing experiment, edge profiles of ablation hole circles C_1_ with diameter D_1_ and C_2_ with diameter D_2_ were clearly observed. HAZ was located between C_1_ and C_2_ with spacing of D_3_, the bump formed after PET melting and solidification. In the line laser processing experiment, edge profiles of ablation kerf lines L_1_, L_2_, L_3_ and L_4_ could be clearly observed. Among them, HAZ above kerf was located between L_1_ and L_2_ with spacing of d_1_. Kerf was located between L_2_ and L_3_ with spacing of d_2_. HAZ below kerf was located between L_3_ and L_4_ with spacing of d_3_. The values of hole diameter (D_H_), processing area diameter of hole (D_PH_), HAZ width of hole or bump (D_HH_, maximum) and kerf width (W_K_) were initialized as the values of D_1_, D_2_, D_3_ and d_2_, respectively. HAZ width of kerf (W_HK_) and processing area diameter of kerf (W_PK_) were calculated using the following equations:

W_HK_ = MAX:(d_1_, d_3_) (1)

W_PK_ = d_1_ + d_2_ + d_3_ (2)

### 2.4. Characterizations

A scanning electron microscope (SEM, HITACHI SU3900, Tokyo, Japan) was applied to observe surface morphologies of the kerf and hole of PET film samples. A thermogravimetry-infrared association meter (TGA-FTIR, PerkinElmer, Waltham, MA, USA) was used for the thermogravimetric analysis (TGA, 25–1000 °C) of PET film, and IR spectra (500–4000 cm^−1^) of gaseous decomposition products of PET film at different temperatures were collected during thermal decomposition process. A pyrolysis–gas chromatography-mass spectrometer (GC-MS, Agilent 7890A/5975C, Santa Clara, CA, USA) was used to analyze gaseous decomposition products of PET film during rapid pyrolysis (25–1000 °C, 50 °C/ms, N_2_). A laser confocal Raman spectrometer (532 nm, 500–3000 cm^−1^, LabRAM HR800, Horiba JobinYvon, Palaiseau, France) was used for the Raman spectra of PET film and its solid decomposition products.

## 3. Results and Discussion

### 3.1. Laser Ablation Mechanism of PET Film

**Figure 2:**
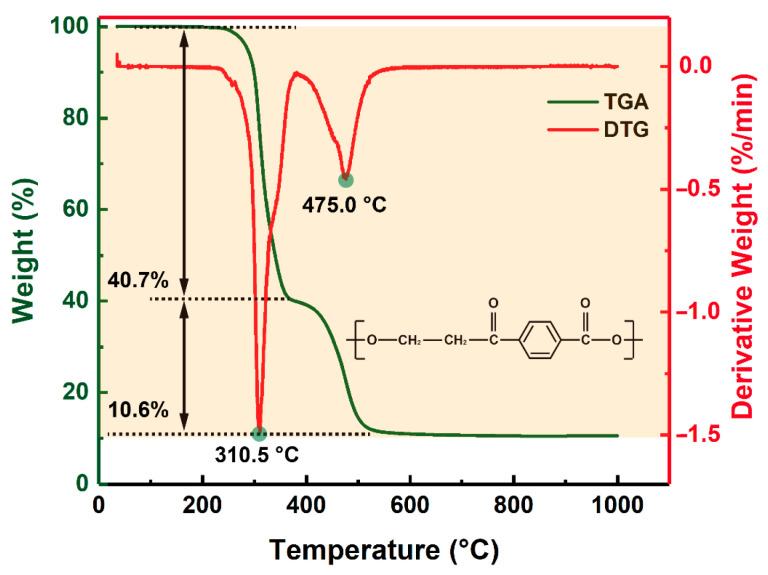
TGA-DTG curves of the PET film.

During the laser processing of polymers, laser ablation is a combination of photothermal and photochemical processes [26,29]. Between these two mechanisms, the photothermal mechanism plays an important role in laser processing, especially for transparent polymers with poor absorption. It is important and effective to analyze the laser ablation products during photothermal ablation in order to understand how the laser ablation of polymers works, as well as improving its quality. Therefore, thermogravimetric analysis was performed to analyze the thermal decomposition process of the PET film. Figure 2 shows the TGA-DTG curves of PET film. The thermal decomposition process of the PET film consisted of two steps. First, when the temperature reached about 310.5 °C, the PET film started to decompose and its weight dropped to about 40.7%. Second, when the temperature reached about 475.0 °C, the PET film further decomposed and its residue weight was about 10.6%.

**Figure 3:**
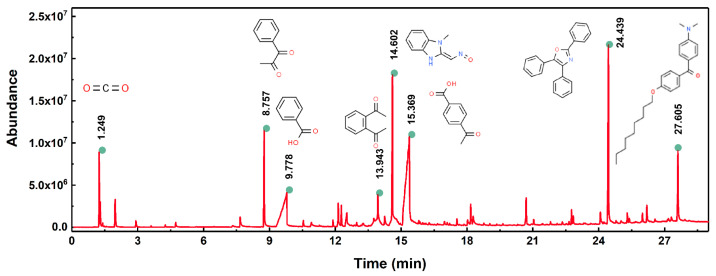
Ion current of evolved gaseous products during pyrolysis of PET film.

After this, pyrolysis–gas chromatography-mass spectrometry was performed to analyze the gaseous decomposition products of the PET film during pyrolysis. Figure 3 shows the ion current of evolved gases during its pyrolysis. The results showed that the gaseous decomposition products produced during the pyrolysis of the PET film were mainly composed of: carbon dioxide (3.90%); 1-phenyl-1,2-propanedione (4.65%); benzoic acid (12.56%); o-diacetylbenzene (3.90%); benzimidazole-2-carboxaldehyde (11.56%); 1-methyl-, oxime 4-acetylbenzoic acid (26.17%); 2,4,5-triphenyl-1,3-oxazole (11.67%); (4-dimethylamino-phenyl)-(4-nonyloxy-phenyl)-methanone (4.28%); etc. Table 3 lists the information about the gaseous decomposition products of the PET film in detail. These results showed that during the pyrolysis process, the PET film either decomposed into small, molecular-like carbon dioxide, or recomposed to form new substances such as 2,4,5-triphenyl-1,3-oxazole. Ambient gases also participated in the pyrolysis process of the PET film. During the laser processing of the PET film, the decomposition process and products of the PET film were more complicated, clearly due to the more complex environment and the more complex interactions between laser, heat and matter.

**Figure 4:**
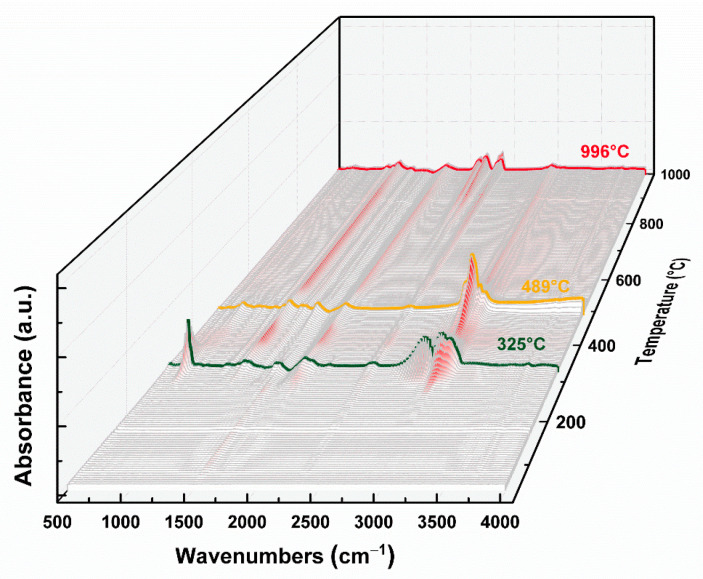
Infrared spectrogram of evolved gaseous products during pyrolysis of PET film.

**Figure 5:**
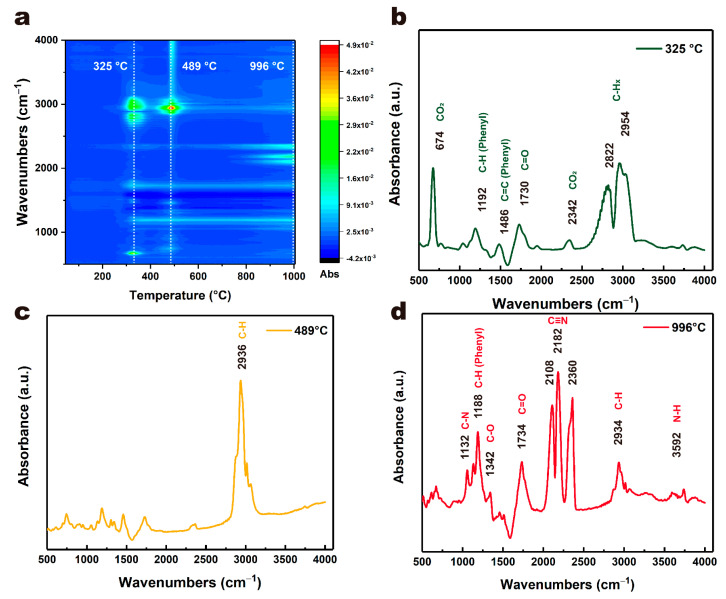
Infrared spectral intensity maps of evolved gaseous products during pyrolysis of PET film (**a**), and infrared spectrogram at typical temperatures: (**b**) 335 °C, (**c**) 489 °C and (**d**) 996 °C.

Furthermore, TGA-FTIR was used to analyze gaseous decomposition products formed during the pyrolysis of the PET film. FTIR spectra of gaseous products at different temperatures from 50 to 1000 °C are presented in Figure 4 and Figure 5. There was no obvious absorption peak below about 300 °C, which meant no decomposition. Above 300 °C, obvious absorption peaks gradually appeared and the intensity reached its maximum at 325 °C and 489 °C (Figure 5a), respectively, which indicated that the PET film began to decompose gradually and was consistent with the thermal analysis results. As shown in Figure 5b, the characteristic peaks of carbon dioxide (674 cm^−1^ and 2342 cm^−1^), phenyl (1192 cm^−1^ for C-H, 1486 cm^−1^ for C=C) and carbonyl group (1730 cm^−1^ for C=O) were observed in the gaseous products at 325 °C, which might be due to the formation of small molecules such as carbon dioxide and benzoic acid. The peaks at 2822 cm^−1^ and 2954 cm^−1^ were assigned to CH_x_ stretching vibrations [30,31]. At 489 °C, a stronger C-H characteristic peak was observed at 2936 cm^−1^. When the temperature rose above 996 °C, the PET film further decomposed and recomposed into small molecules. Here, the characteristic peaks of carbon nitrogen compounds (1132 cm^−1^ for C-N, 2108 cm^−1^, 2182 cm^−1^ and 2360 cm^−1^ for C≡N) and carbon oxides (1342 cm^−1^ for C-O) appeared, but the characteristic peaks of phenyl and carboxyl groups did not, which indicated the PET film not only decomposed, but also recomposed and even reacted with ambient gases under high temperature. These FTIR results at different temperatures were consistent with the pyrolysis results.

**Figure 6:**
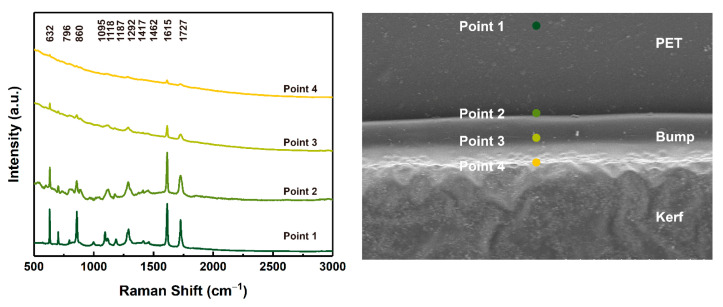
The Raman spectra of PET film after laser processing.

In addition to the gaseous decomposition products, the solid residues after laser ablation were analyzed by a Raman spectrometer. Figure 6 shows the Raman spectra of different areas of the PET film after laser processing: the unprocessed area of the native PET film (Point 1); the outer edge of the bump (Point 2); the surface of the bump (Point 3); and the inner surface of the kerf, or inner edge of the bump (Point 4). The Raman spectrum of the PET film was consistent with previous reports [32], in which characteristic peaks of terephthalate and of ethylene glycol (CH_2_-CH_2_-O) functional groups were found. In detail, the characteristic peaks at 632 cm^−1^ (CCC in plane bending (phenyl)), 796 cm^−1^ (C-H out of plane bending (phenyl)), 860 cm^−1^ (C-C stretching (phenyl breathing), C-O stretching), 1095 cm^−1^ (C-C stretching (glycol)), 1118 cm^−1^ (C-H in plane bending (phenyl), C-O stretching), 1187 cm^−1^ (C-H in plane bending (phenyl)), 1292 cm^−1^ (C-C stretching (phenyl), C-O stretching), 1416 cm^−1^ (C-C stretching (phenyl)), 1615 cm^−1^ (C=C stretching (phenyl)) and 1726 cm^−1^ (C=O stretching) were observed in the Raman spectrum of the PET film (Point 1) [32]. After laser ablation, a kerf, a bump and splashes were observed in the laser processing area due to the melting of the PET film caused by heat. At Points 2, 3 and 4, the characteristic peak intensity of the functional groups in the PET film decreased to varying degrees, and the closer these were to the central laser processing area, the lower their intensity, and in some cases they even vanished. This indicated that during the laser processing of PET film, the polymer chains absorbed enough heat to cause their bonds to break and decompose into small molecular fragments, due to the photothermal mechanism. The closer to the processing center, the higher the temperature and the more complete the decomposition of the polymer chains was.

**Figure 7:**
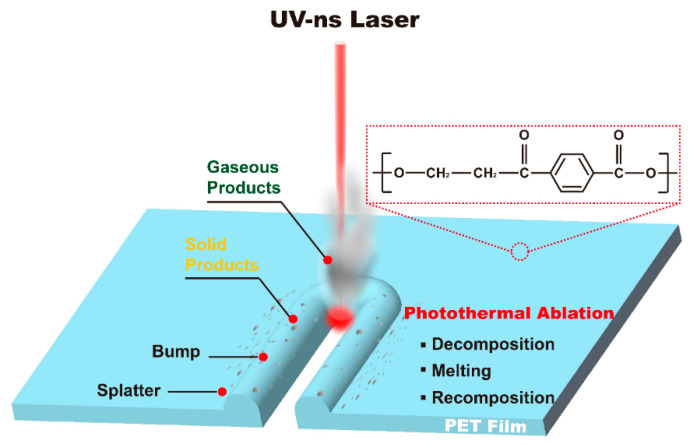
Schematic diagram of the interaction mechanism between the UV-ns laser and the PET film.

According to the above results, showing gaseous products caused by thermal decomposition and solid products caused by laser ablation of the PET film, the laser processing process and mechanism of the PET film were clear. Figure 7 illustrates the schematic diagram of the interaction mechanism between nanosecond UV laser and the PET film during laser processing. Firstly, the PET film was almost transparent at the laser excitation wavelength (355 nm), and the absorbed single photon energy (3.49 eV at 355 nm) was insufficient to break the polymer backbone bonds directly (3.69 eV for C-C). The photothermal mechanism dominated the laser processing or ablation process of the PET film, and the decomposition of the PET polymer chains was mainly pyrolysis [13,26,33,34,35]. Secondly, regarding the laser irradiation and photothermal conversion, the PET polymer chains either decomposed into small molecules or short-chain polymers, or recomposed [13,26,36]. At the same time, ambient gases such as oxygen and nitrogen also participated in this process. Thirdly, as the temperature changed, thermoplastic PET film melted and solidified, resulting in the formation of bumps around the kerf [13], around which the splatter deposited.

### 3.2. The Morphology of Features at Different Temperatures

According to the laser processing mechanism of PET film, it can be seen that the conversion, absorption and transfer of heat as well as the change in temperature had vital influences on this process. Therefore, the external temperature field-assisted laser processing of PET film experiment was designed and performed to investigate the laser processing of PET film at different ambient temperatures and the effects of temperature on the laser processing of PET film, in which single-point laser ablation and line laser ablation were carried out at different temperatures (−25 °C, 0 °C, 25 °C, 50 °C, 75 °C, 100 °C).

**Figure 8:**
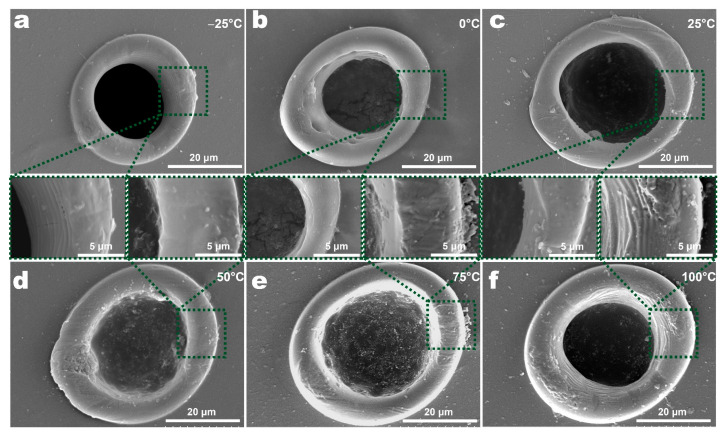
SEM images of holes of PET films at different ambient temperatures in the single-spot laser processing experiment: (**a**) −25 °C, (**b**) 0 °C, (**c**) 25 °C, (**d**) 50 °C, (**e**) 75 °C, (**f**) 100 °C.

**Figure 9:**
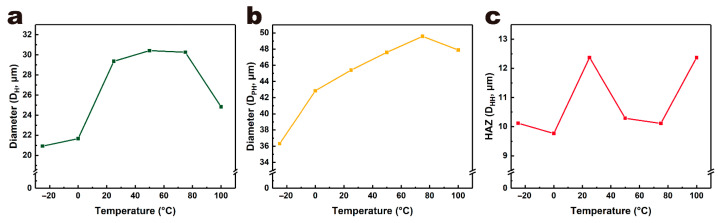
Hole diameter (**a**), processing area diameter (**b**) and HAZ (**c**) of PET films at different ambient temperature in the single spot laser processing experiment.

Figure 8 and Figure 9 show the morphologies and sizes of ablation holes in the single-point laser ablation of PET films at different temperatures. As the ambient temperature increased, the so too did the diameter of the ablation hole and processing area. At room temperature (25 °C), the diameter of the ablation hole and processing area was 29.36 μm and 45.4 μm, respectively. When ambient temperature decreased to 0 °C and −25 °C, the diameter of the ablation hole was 21.67 μm and 20.92 μm, respectively, and the diameter of processing area was 42.85 μm and 36.30 μm, respectively. When the ambient temperature was above room temperature, the diameter of the ablation hole was 30.42 μm at 50 °C, 30.26 μm at 75 °C and 24.84 μm at 100 °C, respectively, and the diameter of processing area was 47.60 μm at 50 °C, 49.57 μm at 75 °C and 47.88 μm at 100 °C, respectively. As for the HAZ or the bump, the size was 10.12 μm at −25 °C, 9.77 μm at 0 °C, 12.37 μm at 25 °C, 10.29 μm at 50 °C, 10.11 μm at 75 °C and 12.37 μm at 100 °C, respectively. The effect of ambient temperature on the size of the HAZ was not obvious. In addition to the change in feature sizes, the morphology of the holes also changed. When the temperature was relatively low (−25 °C, 0 °C, 25 °C), the holes were smooth and clean. However, when the temperature increased (50 °C, 75 °C, 100 °C), wrinkles and debris appeared.

**Figure 10:**
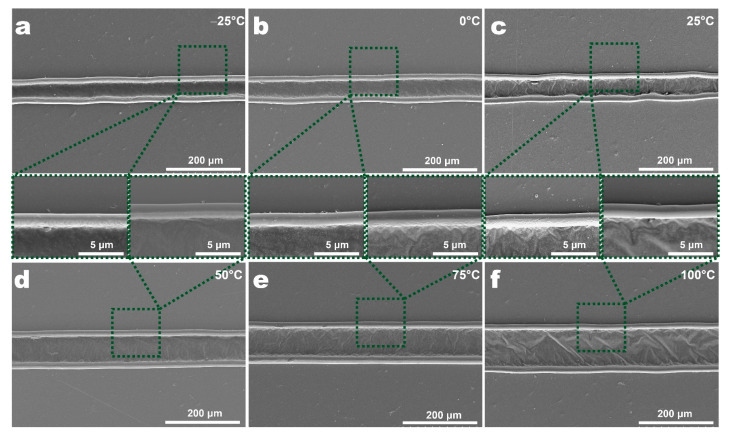
SEM images of kerfs of PET films at different ambient temperatures in line laser processing experiment: (**a**) −25 °C, (**b**) 0 °C, (**c**) 25 °C, (**d**) 50 °C, (**e**) 75 °C, (**f**) 100 °C.

**Figure 11:**
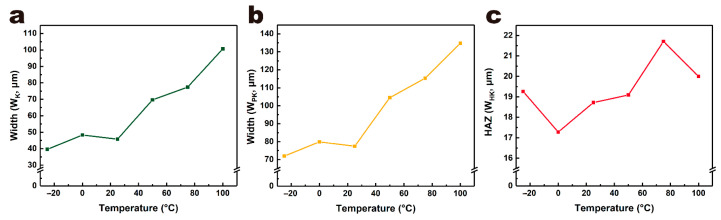
Kerf width (**a**), processing area width (**b**) and HAZ (**c**) of PET films at different ambient temperatures in line laser processing experiment.

Figure 10 and Figure 11 show the morphologies and sizes of ablation kerfs in the line laser ablation of PET films at different ambient temperatures. Similar to the single-spot ablation experiment, as the ambient temperature increased, the width of the ablation kerf and processing area also increased. The kerf width was 39.63 μm at −25 °C, 48.30 μm at 0 °C, 45.81 μm at 25 °C, 69.66 μm at 50 °C, 77.36 μm at 75 °C and 100.70 μm at 100 °C. The processing area width was 71.96 μm at −25 °C, 79.84 μm at 0 °C, 77.43 μm at 25 °C, 104.52 μm at 50 °C, 115.30 μm at 75 °C and 134.73 μm at 100 °C. The HAZ width also showed an approximate slow increase trend with increased ambient temperature. The team systematically studied the influences of laser processing parameters (repetition rate, cutting speed and cutting times) on laser processing of PET film [13]. Although optimizing laser processing parameters could improve laser processing to a certain extent, its effect was very limited. Compared to this, adjusting ambient temperature in the current study improved the laser processing process of the PET film more effectively and significantly, which means that this method possesses greater application potential and scope in actual industrial production.

### 3.3. The Effects of Temperature on Laser Processing of PET Film

Through the above analysis of the morphologies and sizes of the features (hole and kerf), the effects of ambient temperature on the laser processing of the PET film were revealed. Increasing the ambient temperature promoted the pyrolysis of the PET film and improved laser ablation efficiency, so the sizes of the hole, kerf and processing area increased with the increase in ambient temperature. However, when the ambient temperature was too high, this promotion and improvement might have caused the non-uniform and incomplete decomposition of the PET polymer chain, resulting in debris deposited in the processing area. Indeed, low ambient temperature reduced the HAZ and the thermal effect, while the increase in ambient temperature resulted in an increase in the HAZ and thermal stress, which caused the increase of size of the HAZ or bump and the appearance of wrinkles. The root cause of the above effects on the laser processing of the PET film was that the increase in ambient temperature promoted the heat transfer in the laser processing. The above analysis showed that a proper ambient temperature could not only improve the laser processing efficiency of the PET film, but also ensure good quality [36].

**Figure 12:**
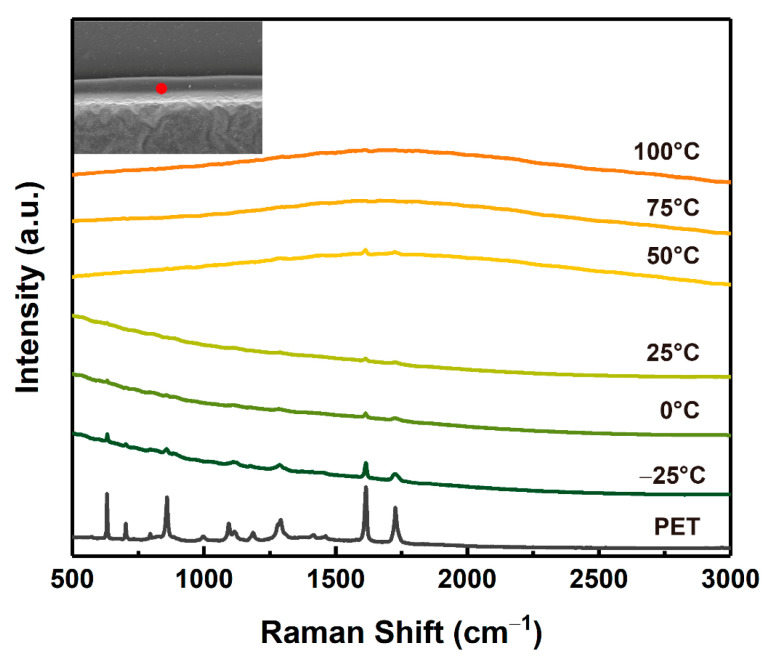
The Raman spectra (at HAZ or bump) of PET films at different temperatures.

In order to verify and study the effects of ambient temperature on the laser processing of PET film further, the Raman spectra (at HAZ or Bump) of PET films processed at different temperatures were acquired, as shown in Figure 12. As the ambient temperature increased, the characteristic peaks of the functional groups and chemical bonds in the PET film gradually decreased or even vanished. When the ambient temperature was low (−25 °C, 0 °C, 25 °C), the peaks at 632 cm^−1^, 860 cm^−1^, 1187 cm^−1^, 1292 cm^−1^, etc., were recognizable. However, when the ambient temperature was above 50 °C, these peaks gradually decreased or even disappeared. The difference in Raman spectra of PET films processed at different ambient temperatures proved that the increase in ambient temperature promoted heat transfer during the laser processing, which promoted the pyrolysis of the PET film and made the HAZ increase.

## 4. Conclusions

The photothermal ablation mechanism and the effects of ambient temperature on the laser processing of PET film were investigated. According to our studies on the pyrolysis of PET film and the temperature field-assisted UV-ns pulse laser processing, the main conclusions are as follows:

1. When the PET film is almost transparent at the laser excitation wavelength (355 nm), and the single photon energy at 355 nm is insufficient to break the polymer backbone bonds directly, the laser processing of PET film is dominated by the photothermal decomposition process (pyrolysis).

2. During the laser processing of PET film, PET polymer chains decompose into small fragments, which recompose, and ambient gases also participate in this process. As the ambient temperature changes, thermoplastic PET film melts, resulting in the formation of a bump. Splatter is deposited in the processing area.

3. An adjustment in ambient temperature affects the laser processing of PET film. An increase in ambient temperature changes the heat transfer and temperature distribution in the laser processing. A low ambient temperature reduces the thermal effect, and an increase in ambient temperature improves the efficiency (kerf width: 39.63 μm at −25 °C, 45.81 μm at 25 °C, 100.70 μm at 100 °C) but exacerbates the thermal effect.

This work provides effective methods to study the laser processing mechanism of polymer films as well as an approach to improve the laser processing of polymer films.

## Figures and Tables

**Table 1:** The specific parameters of laser processing system.

Parameters	Values
Wavelength	355 nm
Pulse width	16 ± 2 ns@50 kHz
Beam diameter	>11.8 μm
Focal length	167 mm

**Table 2:** The experimental design.

Experiment	No.	Temperature (°C)	Parameters
**Single-Point** **Laser Processing**	1	−25	Laser Power = 0.9 W Repetition Frequency = 200 kH_Z_ Duration Time = 0.05s
	2	0	
	3	25	
	4	50	
	5	75	
	6	100	
**Line** **Laser Processing**	1	−25	Laser Power = 0.9 W Repetition Frequency = 200 kHz Scanning Speed = 50 mm/s Scanning Times = 30
	2	0	
	3	25	
	4	50	
	5	75	
	6	100	

**Table 3:** Composition table of the evolved gaseous products during pyrolysis of PET film.

Peak	RT (min)	Area (%)	ID	CAS#	2D	3D
1#	1.249	3.90	Carbon dioxide	000124-38-9	** 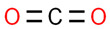 **	** 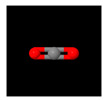 **
2#	8.757	4.65	1-Phenyl-1,2-propanedione	000579-07-7	** 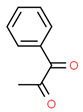 **	** 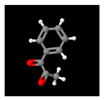 **
3#	9.778	12.56	Benzoic acid	000065-85-0	** 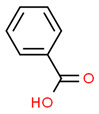 **	** 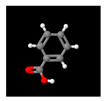 **
4#	13.943	3.90	o-Diacetylbenzene	000704-00-7	** 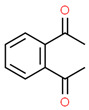 **	** 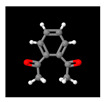 **
5#	14.602	11.56	Benzimidazole-2-carboxaldehyde, 1-methyl-, oxime	003013-07-8	** 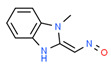 **	** 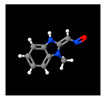 **
6#	15.369	26.17	4-Acetylbenzoic acid	000586-89-0	** 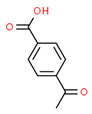 **	** 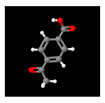 **
7#	24.439	11.67	2,4,5-triphenyl-1,3-oxazole	000573-34-2	** 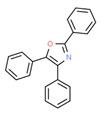 **	** 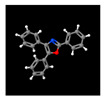 **
8#	27.605	4.28	(4-Dimethylamino-phenyl)-(4-nonyloxy-phenyl)-methanone	300382-52-9	** 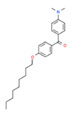 **	** 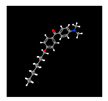 **

## Data Availability

The data presented in this study are available on request from the corresponding author. The data are not publicly available because the data are also part of an ongoing study.
